# Alternative Methods for Grouping Race and Ethnicity to Monitor COVID-19 Outcomes and Vaccination Coverage

**DOI:** 10.15585/mmwr.mm7032a2

**Published:** 2021-08-13

**Authors:** Paula Yoon, Jeffrey Hall, Jennifer Fuld, S. Linda Mattocks, B. Casey Lyons, Roma Bhatkoti, Jane Henley, A.D. McNaghten, Demetre Daskalakis, Satish K. Pillai

**Affiliations:** 1 CDC COVID-19 Response Team.

Population-based analyses of COVID-19 data, by race and ethnicity can identify and monitor disparities in COVID-19 outcomes and vaccination coverage. CDC recommends that information about race and ethnicity be collected to identify disparities and ensure equitable access to protective measures such as vaccines; however, this information is often missing in COVID-19 data reported to CDC. Baseline data collection requirements of the Office of Management and Budget’s Standards for the Classification of Federal Data on Race and Ethnicity (Statistical Policy Directive No. 15) include two ethnicity categories and a minimum of five race categories (*1*). Using available COVID-19 case and vaccination data, CDC compared the current method for grouping persons by race and ethnicity, which prioritizes ethnicity (in alignment with the policy directive), with two alternative methods (methods A and B) that used race information when ethnicity information was missing. Method A assumed non-Hispanic ethnicity when ethnicity data were unknown or missing and used the same population groupings (denominators) for rate calculations as the current method (Hispanic persons for the Hispanic group and race category and non-Hispanic persons for the different racial groups). Method B grouped persons into ethnicity and race categories that are not mutually exclusive, unlike the current method and method A. Denominators for rate calculations using method B were Hispanic persons for the Hispanic group and persons of Hispanic or non-Hispanic ethnicity for the different racial groups. Compared with the current method, the alternative methods resulted in higher counts of COVID-19 cases and fully vaccinated persons across race categories (American Indian or Alaska Native [AI/AN], Asian, Black or African American [Black], Native Hawaiian or Other Pacific Islander [NH/PI], and White persons). When method B was used, the largest relative increase in cases (58.5%) was among AI/AN persons and the largest relative increase in the number of those fully vaccinated persons was among NH/PI persons (51.6%). Compared with the current method, method A resulted in higher cumulative incidence and vaccination coverage rates for the five racial groups. Method B resulted in decreasing cumulative incidence rates for two groups (AI/AN and NH/PI persons) and decreasing cumulative vaccination coverage rates for AI/AN persons. The rate ratio for having a case of COVID-19 by racial and ethnic group compared with that for White persons varied by method but was <1 for Asian persons and >1 for other groups across all three methods. The likelihood of being fully vaccinated was highest among NH/PI persons across all three methods. This analysis demonstrates that alternative methods for analyzing race and ethnicity data when data are incomplete can lead to different conclusions about disparities. These methods have limitations, however, and warrant further examination of potential bias and consultation with experts to identify additional methods for analyzing and tracking disparities when race and ethnicity data are incomplete.

To improve monitoring of COVID-19–associated outcomes among racial and ethnic groups, CDC used three methods for grouping persons by race and ethnicity to analyze the following six indicators: 1) COVID-19 case counts, 2) cumulative incidence, 3) rate ratios for COVID-19 infection, 4) number of fully vaccinated persons, 5) cumulative vaccination coverage rates, and 6) rate ratios for being fully vaccinated. The method for grouping race and ethnicity used by CDC (current method) begins by grouping persons with Hispanic ethnicity as Hispanic, regardless of race, then groups persons with reported race and non-Hispanic ethnicity as race category, non-Hispanic (which excludes persons with missing or unknown ethnicity and those with non-Hispanic ethnicity and missing or unknown race). The current method was compared with two alternative methods (methods A and B) that have been used previously (*2*,*3*). Method A first groups persons based on Hispanic ethnicity (as with the current method) and then groups persons with known race and non-Hispanic ethnicity or unknown or missing ethnicity as race category, non-Hispanic (persons with missing or unknown race and missing or unknown or non-Hispanic ethnicity are excluded). Method B groups all persons with Hispanic ethnicity as Hispanic, regardless of race, and persons with reported race and Hispanic, non-Hispanic, unknown, or missing ethnicity are grouped by race category; persons with missing or unknown race and missing or unknown or non-Hispanic ethnicity are excluded. Notably, with method B, the groups are not mutually exclusive (Box).

BOX Methods for grouping race and ethnicity* for COVID-19 cases, January 1, 2020–May 31, 2021, and fully vaccinated persons, December 14, 2020–May 31 — United States, 2021

**Current method**

**Race/Ethnicity groups**

- American Indian or Alaska Native, non-Hispanic
- Asian, non-Hispanic
- Black or African American, non-Hispanic
- Hispanic
- Native Hawaiian or Other Pacific Islander, non-Hispanic
- White, non-Hispanic

**Grouping method**

1. Persons with Hispanic ethnicity are grouped as Hispanic, regardless of race.

2. For the remaining records, persons with reported race and non-Hispanic ethnicity, are grouped as race category, non-Hispanic.

3. Persons with missing or unknown ethnicity are excluded even if race is reported, and persons with non-Hispanic ethnicity and missing or unknown race are excluded.

**Method A**

**Race/Ethnicity groups**

- American Indian or Alaska Native, non-Hispanic
- Asian, non-Hispanic
- Black or African American, non-Hispanic
- Hispanic
- Native Hawaiian or Other Pacific Islander, non-Hispanic
- White, non-Hispanic

**Grouping method**

1. Persons with Hispanic ethnicity are grouped as Hispanic, regardless of race.

2. For the remaining records, persons with reported race and non-Hispanic, unknown, or missing ethnicity, are grouped as race category, non-Hispanic.

3. Persons with missing or unknown race and missing or unknown or non-Hispanic ethnicity are excluded.

**Method B**

**Race/Ethnicity groups**

- American Indian or Alaska Native
- Asian
- Black
- Hispanic
- Native Hawaiian or Other Pacific Islander
- White

**Grouping method**

1. For all records, persons with Hispanic ethnicity are grouped as Hispanic, regardless of race.

2. Persons with reported race and ethnicity that is Hispanic, non-Hispanic, unknown, or missing are grouped by race category.

3. The groups are not mutually exclusive.

4. Persons with missing or unknown race and missing or unknown or non-Hispanic ethnicity are excluded.

*Multiracial and other race were excluded from analysis.

Daily confirmed COVID-19 cases in the United States during January 1, 2020–May 31, 2021, were obtained from CDC’s case-based surveillance system.* Daily data about COVID-19 vaccine doses administered in the United States during December 14, 2020–May 31, 2021, including full vaccination status, were collected by vaccination providers and reported to CDC by multiple sources.^†^ In the case and vaccination data sent to CDC, race was reported as White, Black, AI/AN, Asian, NH/PI, more than one race, other race, unknown race, or missing race. Ethnicity was reported as Hispanic or Latino (Hispanic), non-Hispanic, unknown ethnicity, or missing ethnicity. COVID-19 incidence and vaccination coverage rates were calculated using the 2019 U.S. Census Bureau’s annual resident population estimates.^§^ The current method and method A used the same population groupings (denominators) for rate calculations (Hispanic persons for the Hispanic group and race category, non-Hispanic persons for the different racial groups). Method B denominators were Hispanic persons for the Hispanic group and persons of Hispanic or non-Hispanic ethnicity for the different racial groups. Rate ratios were used to compare relative differences in COVID-19 incidence and full vaccination coverage rates between racial and ethnic groups. The comparator for the current method and method A was White, non-Hispanic persons and for method B was White persons. This activity was reviewed by CDC and was conducted consistent with applicable federal law and CDC policy.^¶^

During January 1, 2020–May 31, 2021, U.S. states and four territories reported 26,724,149 COVID-19 cases to CDC. Among these reports, information on race, ethnicity, or both was missing from 26.7%, 35.2%, and 21.7% of reports received, respectively. During December 14, 2020–May 31, 2021, based on vaccine administration data reported to CDC, 126,692,891 fully COVID-19–vaccinated persons were reported in the United States; information on race, ethnicity, or both was missing from 23.1%, 31.7%, and 19.5% of these reports, respectively.

Among persons of Hispanic ethnicity, the numbers of COVID-19 cases and persons fully vaccinated, and population incidence and vaccination coverage rates were the same across the three methods for grouping race and ethnicity (Table 1). Methods A and B resulted in more COVID-19 cases and fully vaccinated persons assigned to a racial group compared with the current method because of the inclusion of persons with unknown or missing ethnicity information. Compared with the current method, method A resulted in case counts that were 16.6% to 37.2% higher across race groups, with the largest relative increase in the AI/AN, non-Hispanic group (37.2%). For method B, for which racial and ethnic groups were not mutually exclusive, the percentage increase in case counts compared with the current method ranged from 25.7% to 58.5% among the five race categories. The largest relative increase in case counts was in the AI/AN group (58.5%); case counts in White persons also increased (45.1%). The estimated population incidence of COVID-19 varied depending on the classification method used. Compared with the current method, method A resulted in higher cumulative COVID-19 incidences among the five racial groups, with the largest increase among AI/AN, non-Hispanic persons (37.2%). Method B resulted in increased cumulative incidence among Asian persons (21.8%), Black persons (19.6%) and White persons (14.3%), and slight decreases among AI/AN persons (7.9%) and NH/PI persons (1.0%).

**TABLE 1:** Counts, relative change, and population rates* using three methods for grouping race and ethnicity for COVID-19 cases, January 1, 2020–May 31, 2021 and fully vaccinated persons, December 14, 2020–May 31, 2021 — United States

Race and ethnicity grouping method	No.of COVID-19 cases^† ^(% change^§^ compared with current method)	No. of cases per 100,000 persons (% change^¶^ compared with current method)	No. of fully vaccinated persons**^ ^(% change^§^ compared with current method)	No. of fully vaccinated persons per 100,000 (% change^¶^ compared with current method)
**Current method** ^††^				
AI/AN, non-Hispanic	163,818 (N/A)	6,728 (N/A)	797,443 (N/A)	32,750 (N/A)
Asian, non-Hispanic	543,027 (N/A)	2,872 (N/A)	5,002,826 (N/A)	26,462 (N/A)
NH/PI, non-Hispanic	50,158 (N/A)	8,417 (N/A)	231,611 (N/A)	38,867 (N/A)
Black, non-Hispanic	1,890,813 (N/A)	4,595 (N/A)	7,215,273 (N/A)	17,535 (N/A)
Hispanic or Latino	4,835,843 (N/A)	7,984 (N/A)	10,897,572 (N/A)	17,991 (N/A)
White, non-Hispanic	8,392,146 (N/A)	4,253 (N/A)	52,872,482 (N/A)	26,797 (N/A)
**Total**	**15,875,805 (N/A)**	**N/A**	**77,017,207 (N/A)**	**N/A**
**Method A** ^§§^				
AI/AN, non-Hispanic	224,761 (37.2)	9,231 (37.2)	950,367 (19.2)	39,031 (19.2)
Asian, non-Hispanic	666,250 (22.7)	3,524 (22.7)	5,893,828 (17.8)	31,175 (17.8)
NH/PI, non-Hispanic	58,475 (16.6)	9,813 (16.6)	317,971 (37.3)	53,359 (37.3)
Black, non-Hispanic	2,330,770 (23.3)	5,664 (23.3)	8,575,690 (18.9)	20,841 (18.9)
Hispanic or Latino	4,835,843 (0.0)	7,984 (0.0)	10,897,572 (0.00)	17,991 (0.0)
White, non-Hispanic	10,392,669 (23.8)	5,267 (23.8)	62,750,532 (18.7)	31,803 (18.7)
**Total**	**18,508,768 (N/A)**	**N/A**	**89,385,960 (N/A)**	**N/A**
**Method B** ^¶¶^				
AI/AN	259,591 (58.5)	6,198 (-7.9)	1,047,041 (31.3)	25,000 (-23.7)
Asian	682,590 (25.7)	3,500 (21.8)	5,973,946 (19.4)	30,628 (15.7)
NH/PI	67,275 (34.1)	8,337 (-1.0)	351,176 (51.6)	43,520 (12.0)
Black	2,422,392 (28.1)	5,496 (19.6)	8,838,409 (22.5)	20,053 (14.4)
Hispanic or Latino	4,835,843 (0.0)	7,984 (0.0)	10,897,572 (0.00)	17,991 (0.0)
White	12,175,193 (45.1)	4,860 (14.3)	67,307,494 (27.3)	26,867 (0.3)
**Total**	**20,442,884 (N/A)**	**N/A**	**94,415,638 (N/A)**	**N/A**

**Abbreviations**: AI/AN = American Indian or Alaska Native; N/A = not applicable; NH/PI = Native Hawaiian or Other Pacific Islander.

* Rates for the full period were calculated using the following equation: (cases/population) x 100,000 persons; (fully vaccinated/population) x 100,000 persons. U.S. Census Bureau 2019 single race population estimates were used.

^†^ As of June 7, 2021 (date accessed) CDC’s case-based COVID-19 surveillance system had a total of 26,724,149 reports through May 31, 2021. Persons who were reported as multiracial or other race with non-Hispanic, unknown, or missing ethnicity (1,860,590; 7.0%) were excluded from the analyses.

^§^ Percentage increase compared with current method calculated as ([value method A or B – value current method]/value current method) x 100.

^¶^ Percentage difference compared with current method calculated as ([value Method A or B – value current method]/value current method) x 100.

** As of June 11, 2021 (date accessed) CDC’s vaccine administration surveillance system had a total of 126,692,891 reports through May 31, 2021. Persons who were reported as multiracial or other race with non-Hispanic, unknown, or missing ethnicity (13,859,910; 10.9%).

^†† ^Current method begins by grouping persons with Hispanic ethnicity as Hispanic, regardless of race, then groups persons with reported race and non-Hispanic ethnicity as race category, non-Hispanic; persons with missing or unknown ethnicity and those with non-Hispanic ethnicity and missing or unknown race are excluded.

^§§^ Method A begins by grouping persons with Hispanic ethnicity as Hispanic, regardless of race, then groups persons with known race and non-Hispanic or unknown or missing ethnicity as race category, non-Hispanic; persons with missing or unknown race and missing or unknown or non-Hispanic ethnicity are excluded.

^¶¶^ Method B groups all persons with Hispanic ethnicity as Hispanic, regardless of race, and persons with reported race and Hispanic, non-Hispanic, unknown, or missing ethnicity are grouped by race category; persons with missing or unknown race and missing or unknown or non-Hispanic ethnicity are excluded. Groups are not mutually exclusive.

Compared with the current method, method A resulted in higher numbers of fully vaccinated persons across all racial groups, ranging from 17.8% (non-Hispanic Asian) to 37.3% (non-Hispanic NH/PI) higher. Method B resulted in 19.4% to 51.6% higher numbers of fully vaccinated persons across the racial groups, with the largest relative increase among NH/PI persons (51.6%). Full vaccination coverage also varied depending on the racial and ethnic classification method used. Compared with the current method, method A resulted in higher numbers of fully vaccinated persons per 100,000 for all racial groups, with the largest increase among non-Hispanic NH/PI persons (37.3%). Method B resulted in coverage increases among all racial groups except AI/AN persons, among whom a 23.7% decrease occurred.

When the current method was used, Hispanic and non-Hispanic NH/PI persons were twice as likely as non-Hispanic White persons to have COVID-19 (Table 2). When method A was used, the rate ratio was highest for non-Hispanic AI/AN (1.76) and non-Hispanic NH/PI (1.84) persons; when method B was used, the rate ratio relative to White persons was highest among Hispanic persons (1.72) and NH/PI persons (1.72). Among Asian persons, the rate ratio for COVID-19 was lower across all three methods (0.66–0.71). NH/PI persons had the highest likelihood of being fully vaccinated when the current method (1.70), method A (1.97), and method B (1.92) were used compared with each method’s reference group.

**TABLE 2:** Number of COVID-19 cases, age-adjusted incidence, number of persons fully vaccinated, age-adjusted vaccination coverage, and rate ratios (compared with White persons) using three methods for grouping race and ethnicity for COVID-19 cases, January 1, 2020–May 31, 2021 and fully vaccinated persons, December 14, 2020–May 31, 2021 — United States

Race and ethnicity grouping method	No. of COVID-19 cases* (age-adjusted incidence)^†^	Rate ratio for COVID-19 infection (95% CI)	No. of fully vaccinated persons^§^ (age-adjusted full vaccination coverage)^†^	Rate ratio for fully vaccinated persons (95% CI)
**Current method** ^¶^				
AI/AN, non-Hispanic	163,818 (6,730)	1.59 (1.58–1.60)	797,443 (34,973)	1.41 (1.40–1.41)
Asian, non-Hispanic	543,027 (2,813)	0.67 (0.66–0.67)	5,002,826 (25,625)	1.03 (1.03–1.03)
NH/PI, non-Hispanic	50,158 (8,264)	1.96 (1.94–1.97)	231,611 (42,339)	1.70 (1.70–1.71)
Black, non-Hispanic	1,890,813 (4,616)	1.09 (1.09–1.09)	7,215,273 (19,167)	0.77 (0.77–0.77)
Hispanic or Latino	4,835,843 (8,277)	1.96 (1.96–1.96)	10,897,572 (22,078)	0.89 (0.89–0.89)
White, non-Hispanic	8,392,146 (4,227)	Ref	52,872,482 (24,857)	Ref
**Total**	**15,875,805**	**N/A**	**77,017,207**	**N/A**
Method A**				
AI/AN, non-Hispanic	224,761 (9,187)	1.76 (1.75–1.76)	950,367 (41,709)	1.41 (1.41–1.42)
Asian, non-Hispanic	666,250 (3,442)	0.66 (0.66–0.66)	5,893,828 (30,204)	1.02 (1.02–1.02)
NH/PI, non-Hispanic	58,475 (9,641)	1.84 (1.83–1.86)	317,971 (58,099)	1.97 (1.96–1.98)
Black, non-Hispanic	2,330,770 (5,659)	1.08 (1.08–1.08)	8,575,690 (22,793)	0.77 (0.77–0.77)
Hispanic or Latino	4,835,843 (8,277)	1.58 (1.58–1.58)	10,897,572 (22,078)	0.75 (0.75–0.75)
White, non-Hispanic	10,392,669 (5,228)	Ref	62,750,532 (29,501)	Ref
**Total**	**18,508,768**	**N/A**	**89,385,960**	**N/A**
**Method B** ^††^				
AI/AN	259,591 (6,254)	1.30 (1.29–1.30)	1,047,041 (28,878)	1.11 (1.11–1.11)
Asian	682,590 (3,425)	0.71 (0.71–0.71)	5,973,946 (29,825)	1.14 (1.14–1.14)
NH/PI	67,275 (8,308)	1.72 (1.71–1.73)	351,176 (49,998)	1.92 (1.91–1.92)
Black	2,422,392 (5,517)	1.14 (1.14–1.14)	8,838,409 (22,276)	0.85 (0.85–0.86)
Hispanic	4,835,843 (8,277)	1.72 (1.71–1.72)	10,897,572 (22,078)	0.85 (0.85–0.85)
White	12,175,193 (4,826)	Ref	67,307,494 (26,071)	Ref
**Total**	**20,442,884**	**N/A**	**94,415,638**	**N/A**

**Abbreviations**: AI/AN = American Indian or Alaska Native; CI = confidence interval; N/A = not applicable; NH/PI = Native Hawaiian or Other Pacific Islander; Ref = referent group.

* As of June 7, 2021 (date accessed), CDC’s case-based COVID-19 surveillance system had a total of 26,724,149 reports through May 31, 2021. Persons who were reported as multiracial or other race with non-Hispanic, unknown, or missing ethnicity (1,860,590; 7.0%) were excluded from the analyses.

^†^ Per 100,000 population. Rates were adjusted to the age distribution of the 2019 U.S. Census population estimate.

^§^ As of June 11, 2021 (date accessed), CDC’s vaccine administration surveillance system had a total of 126,692,891 reports through May 31, 2021. Persons who were reported as multiracial or other race with non-Hispanic, unknown, or missing ethnicity (13,859,910; 10.9%) were excluded from the analyses. Texas does not report vaccine counts by race and ethnic group and was excluded.

^¶^ Current method begins by grouping persons with Hispanic ethnicity as Hispanic, regardless of race, then groups persons with reported race and non-Hispanic ethnicity as race category, non-Hispanic; persons with missing or unknown ethnicity and those with non-Hispanic ethnicity and missing or unknown race are excluded.

** Method A begins by grouping persons with Hispanic ethnicity as Hispanic, regardless of race, then groups persons with known race and non-Hispanic or unknown or missing ethnicity as race category, non-Hispanic; persons with missing or unknown race and missing or unknown or non-Hispanic ethnicity are excluded.

^††^ Method B groups all persons with Hispanic ethnicity as Hispanic, regardless of race, and persons with reported race and Hispanic, non-Hispanic, unknown, or missing ethnicity are grouped by race category; persons with missing or unknown race and missing or unknown or non-Hispanic ethnicity are excluded. Groups are not mutually exclusive.

## Discussion

Estimation of COVID-19 incidence and vaccination coverage by race and ethnicity is complicated by missing data. Previous studies have proposed methods for classifying race and ethnicity to address such complexities as multirace responses, but these methods do not consider missing data in circumstances such as a public health emergency in which real-time monitoring and action are needed to identify and address disparities (*4*,*5*). The alternative methods used in this study (methods A and B) resulted in the analyses of more data by race, which increased estimates of COVID-19 case counts, incidence, and vaccination coverage among most racial groups. The current method, used by CDC, and method A resulted in mutually exclusive racial and ethnic groups. The denominators for rate calculations are either persons reported as Hispanic or persons reported as a race category and non-Hispanic, with an assumption in method A that persons for whom missing ethnicity data were missing are non-Hispanic. Method A is more commonly used when ethnicity is missing from a small percentage of records and other information in the record supports a non-Hispanic designation. When approximately one-third of records are missing ethnicity, as in this report (35% for case and 32% for vaccination coverage data), that assumption might attenuate or amplify disparities for certain groups. With method B, the race and ethnicity groups are not mutually exclusive. This complicates comparisons that use a reference group (often White persons), because the race and ethnicity categories overlap.

The findings in this report are subject to at least four limitations. First, because the analysis did not include persons who identified as multiple races or other race, conclusions cannot be drawn about the use of the alternative methods for grouping and analyzing these racial categories. Second, this report did not explore all possible analytic methods for grouping race and ethnicity. For example, imputation (i.e., replacing missing data with other values) has been examined as a potential method to improve estimates of COVID-19 racial and ethnic disparities (*6*). Third, data shared with CDC might undercount COVID-19 cases and vaccination coverage and this undercount might differ by race or ethnicity. Finally, although progress has been made to incorporate the Office of Management and Budget standards (such as Statistical Policy Directive No. 15) into the collection and presentation of race and ethnicity data, some data collection efforts still do not fully use this guidance (*7*).

Although race and ethnicity are not the only measures for assessing health disparities, these measures have been integral to CDC’s understanding of the health outcomes associated with COVID-19 (*8*–*10*). This analysis demonstrates that alternative methods for analyzing race and ethnicity data when data are incomplete can lead to different interpretations about disparities and highlights the importance of working with experts to identify methods for analyzing and tracking disparities when race and ethnicity data are incomplete. CDC uses multiple data sources to monitor disparities in COVID-19 outcomes and will continue to optimize the available data and work with jurisdictions to strengthen reporting of these data consistent with CDC’s COVID-19 Response Health Equity Strategy ** and Data Modernization Initiative.^††^

**Summary**

**What is already known about this topic?**

Analyses of race and ethnicity in COVID-19 data to identify and monitor disparities are complicated by missing or unknown data.

**What is added by this report?**

Methods that use more race information when ethnicity information is missing resulted in higher estimated COVID-19 case counts, incidence, and vaccination coverage for most racial groups studied; however, these methods have limitations and warrant further examination of potential bias.

**What are the implications for public health practice?**

Ongoing work with experts is needed to identify methods for optimizing race and ethnicity data when data are incomplete. Multiple data sources are needed to monitor disparities and continued efforts are needed to strengthen the reporting of these data, consistent with CDC’s Data Modernization Initiative.

## Footnotes

* All 50 states, the District of Columbia, New York City, and four U.S. territories (Guam, Northern Mariana Islands, Puerto Rico, and U.S. Virgin Islands) electronically submit standardized information for individual cases of COVID-19 to CDC via a case report form developed for the CDC COVID-19 response (https://www.cdc.gov/coronavirus/2019-ncov/php/reporting-pui.html) or via the CDC National Notifiable Diseases Surveillance System (https://www.cdc.gov/nndss/action/covid-19-response.html).

† COVID-19 vaccine administration data are reported to CDC by multiple entities using immunization information systems, the Vaccine Administration Management System, pharmacy systems, or direct submission of electronic health records. (https://www.cdc.gov/coronavirus/2019-ncov/vaccines/distributing/about-vaccine-data.html). CDC counts persons as being fully vaccinated if they received 2 doses on different days (regardless of time interval) of the 2-dose mRNA vaccine series or received 1 dose of a single-dose vaccine.

§
https://www.census.gov/data/tables/time-series/demo/popest/2010s-national-detail.html

¶ 45 C.F.R. part 46.102(l)(2), 21 C.F.R. part 56; 42 U.S.C. Sect. 241(d); 5 U.S.C. Sect. 552a; 44 U.S.C. Sect. 3501 et seq.

**
https://www.cdc.gov/coronavirus/2019-ncov/downloads/community/CDC-Strategy.pdf

††
https://www.cdc.gov/surveillance/surveillance-data-strategies/data-IT-transformation.html
